# Dr. Roberton's Additional Remarks on Stricture

**Published:** 1809-07

**Authors:** 


### Dr. Roberton's additional Remarks on Stricture.

(Continued from vol. xxi. pp. 321—332.)

Mr. HOME, near the beginning of his first volume, endeavours to prove the muscularity of the membrane of the urethra, but seems obliged to take a great deal for granted. Many of his speculations on this part of his subject are certainly objectionable; but as they have no strict relation to my present practical inquiries, I shall not now enter into an examination of them. I may however observe, that if we possessed a completely contractile power of the membrane of the urethra, paralysis of the neck of the bladder would not cause incontinence of urine; since, by the contraction of the urethra, it might be retained. We have then no proof whatever of this membrane possessing any contractile power; and the proofs Mr. Home has attempted to give of this are insufficient, viz. the state of it during the passing of urine and semen, and the effect produced on it by the use of injections in gonorrhœa: for, while the urine passes, the muscles of the penis are generally flaccid, and consequently allow the membrane to be easily distended; but, during the emission of semen, these muscles, being suddenly and violently contracted, compress the membrane lining the urethra, and thus it is, at that time, rendered narrower; and the same effect is produced on these parts during the use of injections. Mr. Home constantly runs into error in this way, as if not aware that the muscles have *any* effect in compressing the urethra.—Having presumed the muscularity of the membrane of the urethra, he proceeds to give the following account of the influence of that membrane in the production of stricture.

"Contraction and relaxation," says he, (vol. i. p. 19) "are the natural and healthy actions of the urethra; but this membrane, like every other [part of] muscular structure, is liable to a spasmodic action, which produces a degree of contraction beyond the natural; and in that state the canal loses the power of relaxing till the spasm is removed. When this happens it constitutes disease, and is termed a spasmodic stricture.

"While a stricture is in this state, it is only a wrong action of the membrane of the urethra; and if the parts could be examined in their relaxed state, there would be no appearance of disease.

"When a portion of the urethra is disposed to contract beyond its natural easy state, this disposition commonly increases till the part becomes incapable of falling back into a state of complete relaxation, and the canal remains always narrower at that part.

"In this stage it is both a permanent stricture and a spasmodic one. It is so far permanent, that it is always narrower than the rest of the canal; and so far spasmodic, that it is liable to contract occasionally in a still greater degree."

This, in all probability, is the most correct account of the formation of strictures that can be given; indeed, I believe it is in this way that almost every stricture is formed; and that it is only by neglect of them in their early stages, or by harsh and unwarrantable treatment, that they ultimately become of a permanent nature, or require the complete destruction of their substance, before they can be removed; and even after all this, the patient is often not relieved, but obliged to drag out a life of perpetual misery.

Mr. Home himself seems of this opinion in page 23. Speaking of spasmodic strictures, and their effect in the production of permanent ones, he says, "A stricture having arrived at that stage which renders it permanent, does not prevent it from having also a spasmodic contraction. This however, in many instances, is in a less degree after the disease has been of some years continuance, than at a more early period; for we find patients who have been subject to occasional suppressions, afterwards entirely free from them, the disease in its increase having rendered the parts more indolent, and therefore not so readily affected by accidental causes; but when the stricture becomes very small, the occasional suppressions return, and become more serious." To this I would answer, they certainly do; but under the above circumstances, had proper precautions been used, no such serious termination need have been feared. In short, to sanction the frequent use of the caustic, it is absolutely necessary to admit of more or less spasmodic action in all cases, where what is termed permanent stricture is found. This is indeed Mr. Hunter's opinion, but differently moulded by Mr. Home. If by any one it be asserted, that permanent stricture exists, and if, at the same time, he wishes only to prove the existence of spasmodic stricture, it may be urged that it is purely of that nature; and if, on the contrary, it be contested contested that it is purely spasmodic, it may be admitted that spasm does exist, but that it is in consequence of a permanent obstruction of the canal, which can only be relieved on the removal of the obstruction.

To prevent repetition, I wish it to be particularly understood, that it is to Mr. Home's practice alone that I object; and of his theories, I shall take notice only when they seem likely to lead to erroneous practical conclusions. Mr. Home, in page 31, makes the following remark. "It will appear evident, that a contraction of any particular part of the canal may be brought on by an unusual or preternatural degree of action in the membrane itself, without any new formation whatever."

It need hardly be stated, that in order to form a contraction of any natural canal, possessing, as I have endeavoured to show, no muscular power, additional substance is absolutely necessary.

"A gentleman," says Mr. Home, in page 52, "in the act of copulation, felt, at the instant the emission should have taken place, considerable darting pain in the urethra, and found afterwards a few drops of blood upon his linen; about an hour after he had occasion to make water, and in preparing to do so, the semen, which should have been emitted, appeared upon his shirt in considerable quantity.

"I was consulted upon the cause of such very unusual and distressing circumstances. On hearing them stated, I informed him that there must be a stricture in the urethra, which *alone* could explain what had happened; this he was inclined to doubt, as he made water very well; but upon passing a bougie, an obstruction was met with just beyond the bulb of the urethra, and upon allowing the bougie to remain with slight pressure against the stricture for a few minutes, it was capable of being passed on to the bladder." Now, all these circumstances are more easily explicable, without supposing stricture. During the act of emission, rapid contraction happened in the sphincter vesicæ, which closed the apertures of the vasa deferentia, and when relaxation occurred to allow the passage of the urine, the semen also escaped; and it is in the same manner that semen is often emitted in great quantity immediately after the orgasm. When the bougie was introduced, it had stopped at a small eminence, situated at the under side of the neck of the bladder, which often interrupts the introduction of the staff, and having by pressure overcome this, the bougie entered the bladder. Thus the gentleman himself probably judged correctly that he really had no stricture.

In page 56, a case is related, to show the similarity between stricture and gonorrhœa; this seems to be a case neither of stricture nor of gonorrhœa, but simply a temporary spasmodic affection, with which people advanced in life are often affected when they worship the Cyprian goddess.

In page 62, Mr. Home informs us, that strictures cause fatal peritonitis; and this he thinks extraordinary, as there is no immediate communication between the bladder and abdomen. Is not the bladder covered by the peritonæum? And is not absolute contact immediate communication?—It would appear that gentlemen may, for a moment, forget the most obvious facts of anatomy, while they are absorbed in the search after muscles in the crystaline lens.

To proceed. I cannot find, in all Mr. Home's detail of local symptoms of stricture, a single one which I have not seen in cases purely of a spasmodic nature, and which, when attended to in time, I am daily in the habit of removing by the properly regulated use of internal medicines, external applications, and the occasional use of a simple bougie.

The constitutional symptoms of stricture, mentioned by Mr. Home, are principally of a febrile nature, attended with cold, hot, and sweating stages. In respect to this part of the subject I may remark, that I have seldom met with a very violent case of spasmodic stricture, in which, at some period or other of the disease, these occurrences did not take place. I must however admit, that I have never met with the different stages following each other so accurately as those in which Mr. Home has observed these appearances. All this I conceive to be of little importance, and it can surely never be adopted as a diagnostic symptom of the affection. I think these fits of ague, as they are called, may be accounted for in a more rational way. 1st. From matter being lodged about the perinæum, in consequence of previous improper treatment of the affection, which, assisted by the dread into which the patient is thrown by the very thought of having a caustic bougie passed into his urethra, will occasion the cold fit; and, 2dly, from the pain occasioned by the actual application of a caustic bougie wedged into the stricture.

With

With respect to the case of stricture, treated as an ague, in p. 66, I think Mr. Home's conclusion erroneous. He says, "I have had a patient under my care, who for three years had this constitutional symptom of stricture, in the West Indies, which was treated in that country as an irregular ague; but not finding himself relieved, he came to this country; and it was discovered (by Mr. H.) that he had strictures in the urethra; upon the removal of which, the ague disappeared, without the use of any internal medicine."

From Mr. Home's mode of relating this case, it is evident that he had no symptom to warrant him to suppose the existence of stricture in the urethra, except those usually found in cases of ague. When we consider that, in all probability, the gentleman lived, while in the West Indies, in some situation where ague might be an endemic disease, and that on his return to England he was removed from the very cause of his complaint, in a few weeks, might he not have been entirely freed from it, without either medicine or caustic?

Mr. Home tells us in another place, that strictures have been mistaken for nervous fever; that they cause inflammation of the tonsils, fauces, &c. In short, it would appear that Strictures cause all the diseases that ever either had existence or were invented by Nosologists.

The cases of irritable urethra, mistaken for stricture, (p. 75) according to the account given of them, appear much better fitted for the application of caustic than many of the cases related in the book. Accidentally, however, a simple bougie was preferred, and proved successful. An injection of oil with a small proportion of tinct. opii might have answered the same purpose.

What is the nature of the action produced by the application of the caustic in those cases of irritable urethra related by Mr. Home, where even he allows that no stricture exists? It must be by an entire destruction of the parts that have become morbidly irritable, and are incapable of resuming their healthy action by any power they themselves possess; or it may not entirely destroy the parts, but only cause an alteration of a more healthy nature to take place in them, by which they are rendered capable of performing their natural functions. To effect this last purpose, without running the risk of entirely destroying the organization of the parts, at least deserves some attention, and I have no doubt of our being able to accomplish our object.

The very earliest stages of stricture, before it has acquired that condensed form which nothing but the application of caustic or an instrument can remove, is certainly the time when surgeons ought to be most active and vigorous in their exertions for its removal. I do not hesitate to say, that it is in these moments a surgeon shows whether he is actuated by the powers of discrimination, by which his applications shall be external or internal, as the particular circumstances of the moment shall require; or if his sole dependence is on one remedy, viz. the lunar caustic, by which indeed he may effect a cure, but in doing so may run many chances of involving his patient in great misery perhaps for the remainder of his life.

Mr. Home, in page 109, in a note, informs us of a physician in London, who certainly carried his aversion to the use of caustic, in the removal of permanent stricture, too far. He was probably as much wedded to one side of the question as Mr. Home to the other. The physician, it is said, left his fortune to a nephew, solely on condition that, altho' the uncle knew him to be affected with strictures, he was not to undergo the burning process for their removal. The nephew complied with these conditions, and Mr. Home says he afterwards died in great misery. "In this case," says he, "it was found after death that the urine, prevented by the stricture from coming forward, had forced its way backward upon the intestine, instead of coming through the perinæum, and the first symptom that gave alarm was that of the fæces coming through the penis with the urine."

By what way urine, prevented from being evacuated in the natural way, could be forced backward into the intestine, and the passage which thus conducted it back, enable not only it but even the fæces to overcome the stricture, and pass off by the penis, is not perfectly clear.

In the very early stages of stricture, while yet there remains a great degree of spasm on the parts, and when, in a great majority of cases, the bougie cannot be introduced without occasioning the most excruciating pain, Mr. Home talks with as much coolness of the *irritation,* as he terms it, which is often brought upon the strictured part, as if he were not aware that he is introducing an instrument in this unprepared state into a canal which, from its morbid state, is so susceptible of the most acute sensation. In page 110, he condemns the principal means, viz. internal medicines, by which these effects were to be obviated, ated, because, according to his theory, "No internal medicine appears capable of stopping the progress of a stricture." But, and I do not state it in support of any particular theory, I am in the constant habit, especially if applied at the commencement of the affection, by the *properly regulated* use of internal, and the *properly regulated* employment of external applications, of entirely and permanently removing affections of the most violently spasmodic nature, which in all probability would have terminated in permanent strictures. In short, under the existence of these contractions, no man is warranted to use the bougie, without having previously employed other means. According to Mr. Home, the simple bougie, for the distention of contracted parts, is only of temporary benefit; and in the way he proposes, this was the best effect which could be expected to arise from such practice.

That Mr. Home has acquired from practice a degree of dexterity in the application of the caustic, no one will question; and where strictures of a permanent nature actually do exist, I believe there is scarcely any other surgeon who would be so often successful in their removal. But when it is recollected that it is not the manual dexterity alone of one or even two individuals that can establish a doctrine on scientific principles; and that Mr. H. in detailing his own successful treatment of permanent strictures, is addressing himself to many other persons, it is scarcely to be calculated what an immense number of blunders may be made, not only in mistaking the actual existence of stricture, but in the deficiency of others in their dexterity of applying the caustic for its removal. The seat of the disease, as related by him, the nature and shape of the canal, and the uncertainty of the number and site of these strictures; together with the impossibility, in the hands of many, of applying the caustic bougie to the exact spot where the obstruction lies, as well as the probability, considering the escarotic nature of caustic, of destroying every substance to which it is opposed, ought at all times to induce an exertion to prevent a complaint arriving at that pitch of severity when such applications may be necessary, and to occasion a pause before it is applied, when the propriety of those measures cannot actually be demonstrated.

In former periods, the most commonly preferred plans of operating for the removal of permanent stricture, formidable midable as they may appear to some, were, considering the circumstances I have stated, more certain of effecting the exact object which the surgeon had in view, than the modern attempt to apply caustic through a long narrow passage such as the urethra, where nearly the whole extent of the healthy part of that canal may be cauterized as effectually as the part intended actually to be destroyed.

The most approved of these operations were, dissecting down upon the strictured part and cutting it out; the other by making an opening rather anterior to the stricture, and passing a flexible gum catheter through the opening into the bladder. Even Mr. Home allows, that he has frequently seen this operation successfully performed by John Hunter, and no untoward symptom occur.

From this Mr. Home observes, p. 130, that "if the membrane of the urethra, when diseased, is capable of suffering so much injury, without any consequent symptoms of irritation, it cannot be doubted that it will bear with impunity to be touched *in a very partial manner*, several different times with lunar caustic." But shall we say because a man can, without exhibiting signs of the utmost torture, suffer a cut to be made in his finger, that he will bear with impunity to have his whole hand seared over with a red hot iron! The metaphor is just, for according to the present mode of cauterizing the urethra, I have no idea of a partial application of caustic to that part, and it is absolutely disgusting to hear it talked of. Seven inches, or according to the common language, seven and a quarter, must, less or more according to the dexterity of the operator, be injured by such application. If it receives not much mischief from removing the one nearest the orifice, it must be absolutely destroyed before the removal of the second, third, fourth, fifth, or God knows how many more, which are everlastingly found snugly situated nearer the bladder.

In the following paragraph, p. 131, Mr. Home is certainly right, when he informs us that "his observations are published with a view to extend the use of the caustic to a greater variety of cases, and, in some measure, upon a very different principle from that upon which it was applied to impervious strictures by the late Mr. Hunter." Further on, he says, "he wishes to place the merit of the invention, as well as the mode of applying it, where it was due," viz. to Mr. Hunter; a name which, by the bye, Mr. Home introduces on every occasion where he is in want want of respectable authority to support a repulsive doctrine. I wish Mr. Hunter were alive still to give us his opinion.

I really find so much may be said respecting Mr. Home's practice in stricture, that I fear my readers may imagine I do it from pique; but I assure them this is not the case. I have no knowledge of Mr. Home but from his writings; I have no interest in his success or want of success. Matter of fact and sound reasoning are what I have always been in pursuit of; and I am sorry, for my own ease, I cannot think I have found enough of it in many, even of the best medical publications. But to the point.—In p. 132, Mr. Home, not contented with applying caustic for the removal of permanent strictures; as he calls them, strenuously recommends its use, in preference to the bougie, in what he terms irritable strictures; in other words, I suppose, he means spasmodic strictures. I trust I do not misunderstand him, for I have no wish to do so; however, if I do I shall be glad to be put right. I hope Mr. Home succeeded in all the cases of this nature in which he attempted the introduction of the caustic; I say, I hope so, for if such strictures resembled those of the same kind which are constantly occurring in practice in this place, there must be a peculiar dexterity in his mode of applying that substance so as to cure them. I formerly stated that, in such cases, internal medicines, external applications, and the occasional and most judicious use of the simple bougie, were all in their turn necessary to effect this purpose; that burning away such strictures was merely removing the effect of the spasm for a short period; for the cause still continuing, the constriction would again and again occur; not probably in the same place, to which the caustic was formerly *intended to be applied*, but to every part of the urethra, and possibly to eight or ten different parts of it at the same time, to which it actually was applied.

Mr. Home gives two long cases in proof of this part of his doctrine. He passed bougies repeatedly into the urethra of both patients till he brought on the most alarming symptoms, among which were abscess in perinæo, when he deemed it prudent to desist. He then applied the caustic a variety of times, in each instance, and after occasioning great distress to both patients, he cured them.

In the cure of diseases in general, and of stricture probably among the rest, there is usually, for days or weeks previous to the complete cure, a gradual approach toward that that state. But in not a few of Mr. Home's cases, we find the patient perpetually racked with the most agonizing pains, either from the nature of the disease or from the peculiar quality of his instruments of *cure*; and before we have had a moment to recover from sympathizing in the wretched patient's sufferings, we find him *dismissed cured!*

Mr. Home then, p. 150, enters into a long apologetical oration, with an attempt, at the same time, to show (from instances of other parts of the body suffering violence, without danger) with what safety caustic may be applied to the whole internal membrane of the urethra.

He says, "spasms in particular muscles, as in the intercostalis, diaphragm, muscles of the arm or leg, come on from slight constitutional irritation, or local injuries, attended with little violence; the cause is often so slight as entirely to escape discovery, and the treatment most generally found to succeed, is blistering the surface nearest the part affected, which is one of the most violent applications we are enabled to employ." Had Mr. Home transferred the same sort of reasoning, and the same practice, to those strictures in the urethra of a spasmodic nature, with the judicious addition of other articles which, taken internally or applied externally, might tend to produce similar effects—had he done so, I say, much unnecessary suffering might have been avoided by some who have had courage to submit to such rarely necessary practice, as the burning of the internal membrane of the urethra. Indeed, the application of caustic bougies to the internal membrane of the urethra in these strictures, acts, in their removal, on the same principle as a blister applied to the perinæum or under part of the penis; only with this difference, that in the first, the membrane of the urethra, and much damage to the canal in general, must be the consequence; while in the other, viz. blistering externally, aided by internal medicines, &c. the stricture is removed without doing the slightest degree of injury to these parts. In p. 155, even Mr. Home seems sensible of this, for he says, "this general principle of spasmodic affections and local irritations, yielding more readily to stimulating applications, is now found equally applicable to affections in the urethra." I agree with him; we only differ in our mode of applying them. He burns off the very structure affected, and at least, every part anterior to it, with lunar caustic; while I endeavour to remove it by antispasmodics, blisters, the occasional use of the bougie, &c. without injuring the structure at all. No doubt can be entertained which is the easiest siest and safest way; and there is no one capable of reasoning, but must be convinced of the permanent benefit of the latter, in preference to the former.

In p. 158, Mr. Home commences a comparative inquiry respecting the bougie and the caustic. He says, "it is not my intention, by any means, to discourage the use of the bougie, which is certainly a very useful instrument; *but* as it is found to be limited in its powers, it becomes important to point out a more active application, which may be capable of producing a cure, where that shall have proved inadequate." On the contrary then, I should, according to his mode of using the bougie, entirely discourage it, as, when applied in that way, it is not only very limited in its powers of relief, but productive of the most exquisite distress to the patient; yet, when more scientifically applied, and that in the early stage of stricture, it is preferable, and will ultimately perhaps be preferred to the lunar caustic.

The remaining parts of this section are employed in reasoning upon the comparative effects of these applications. I think it no compliment to Mr. Home, when I state that he reasons on this subject with much accuracy; but he seems in his practice to forget to distinguish the very circumstances upon which his reasonings turn.

In p. 173, Mr. Home informs us, that "it often happens that when there are several strictures, the application of the caustic to that which is nearest the external orifice affects all the others, and makes them relax; so that the stream of urine which before had been very small, shall now be large and free; and after this stricture has been destroyed, and the caustic is applied to one nearer the bladder, the very contrary effect is produced." This he calls sympathy between parts. That sympathy between parts may cause a slight constriction or relaxation of them appears to me very evident; but how, in such a canal as the urethra, it should cause so complete a relaxation of the constricted part as to allow the stream of water before small, to become "large and free", is beyond my comprehension. Pemanently constricted parts become, in some measure, like natural cavities, which force or continued pressure may enlarge to a great extent; but sympathy seldom, I may say never will; particularly in parts such as the urethra, destitute of muscularity, and over which the will has no power. The truth of the matter is just this, the reasoning of Mr. Home can only be applied to a *spasmodic* affection of the part altered in its mode of action fo for the moment by the caustic application, which also caused all those spasms nearer the bladder to disappear. The contrary effect produced, as asserted by Mr. Home, when the caustic is applied to one still nearer the bladder, can never be accounted for by any process of reasoning with which at least I am acquainted: it must have been owing merely to some accidental occurrence. That many curious facts must have occurred to Mr. Home in his practice I have no doubt, but no person should establish a doctrine upon an anomalous fact.

In perusing the 196 pages of Mr. Home's first volume, it has been observed, that numerous spasmodic strictures have by him been considered as permanent, and treated accordingly; and even where caustic is recommended for their removal in preference to the bougie, it may surprize some to find under the head of "circumstances under which the use of the caustic has proved unsuccessful," spasmodic stricture.—Spasmodic stricture then may be incurable by the caustic! Spasm of these parts may be subdivided into six or eight different kinds, and complicated in a variety of ways with permanent stricture, but will this be satisfactory to such readers as chuse to reason for themselves?

Some observations may seem of a paradoxical nature in Mr. Home's works. It is asserted in various parts, and cases detailed to prove it, that the application of caustic for the removal of stricture does not bring on irritation; in other instances, as in page 196, we find that it did bring on irritation. But, perhaps, during the composition of the book, Mr. Home's cases of different result may have occurred, which shows the danger of making statements too general.

To make remarks on all Mr. Home's cases that follow this part of his book, would be unpleasant to myself and uninteresting to my readers; I shall therefore particularly refer to or detail only a few, to which he has given the name of permanent stricture, or such as he deemed only curable by means of the caustic. In this very dry part of his subject, I may have overlooked a number of cases to my purpose; but I believe those I shall subjoin, will be found quite satisfactory in respect to what, in the previous pages, I have endeavoured to prove.

( To be Continued. )

